# A Medical Contract in Italy

**Published:** 1879-08

**Authors:** 


A Medical Contract in Italy.—A singular case of homicide was lately tried at Spoletti, Italy. The accused was a rich farmer, named Carlo Maronni, and his offense was shooting a doctor who had failed to cure his child. When the child, a boy of fourteen months, was taken sick, the farmer sent for the doctor, and proposed to him that if the child recovered he would pay 2,000 lires, (about $400) but if the child died, he should exact the doctor's life. The impecunious doctor accepted the proposition, and an agreement was duly entered into. Unfortunately for both doctor and child, the latter continued to grow worse, and finally died. A few days after, the father exacted his bond, and laying in wait for the doctor, he shot him. The agreement was produced on the trial, and seems to have been considered by the court, who imposed a sentence of only ten years' imprisonment and a fine of 25,000 lires.—*Mich. Med. News.*

